# Associations Between Sound Levels and Sleep Architecture: Implications for Sleep Disparities

**DOI:** 10.1007/s40615-025-02607-2

**Published:** 2025-08-15

**Authors:** Swaty Chapagai, Tâmara P. Taporoski, Erika Yamazaki, Kristen L. Knutson

**Affiliations:** 1 Feinberg School of Medicine, Northwestern University, Chicago, IL USA; 2 Harvard T.H. Chan School of Public Health, Harvard University, Boston, MA USA; 3 Center for Sleep and Circadian Medicine, Department of Neurology, Northwestern University Feinberg School of Medicine, 710 N Lakeshore Drive, Room 1003, Chicago, IL 60611 USA

**Keywords:** Polysomnography, Sleep architecture, Disparities, REM sleep, Environment, Social factors

## Abstract

Poor sleep is more common among some social groups (e.g., minoritized race or lower socioeconomic status). One potential source of sleep disruption is noise. Our goal was to examine whether sound levels varied between two racial groups and education level and whether sound levels were associated with sleep architecture in a real-world setting. Sound was measured using a sound meter while simultaneously recording polysomnography for one night in participants’ bedrooms to quantify sleep stages, non-rapid-eye movement (NREM), and REM sleep. Our sample included 26 Black and 28 White adults aged 21–50 years; 22 (41%) had > college degree. On average, sound levels were higher in the bedrooms of Black participants compared to White participants. Black participants also had significantly less total sleep time, N3, REM, REM%, and higher N2%. Those with college degree or less had lower N3%. Higher average sound levels during the entire sleep period were significantly associated with fewer minutes of REM (*β* = -1.50, *p* = 0.008), lower REM% (*β* = -0.32, *p* = 0.002), and higher N2% (*β* = 0.43, *p* = 0.007) but was not associated with other polysomnography measures. Sound levels were estimated to explain 14.5% of the difference in REM minutes and 21.1% of the difference in REM percentage between races and 6% of the difference in N3% between education groups. In summary, greater sound levels were associated with less REM sleep and greater N2%, and average sound levels were higher in the homes of Black participants. Environmental noise may be one factor associated with sleep disparities.

## Introduction

Numerous prior studies in adults have reported worse sleep health in Black adults compared to White adults in the US. Racial disparities in sleep include sleep architecture, which is comprised of non-rapid eye movement (NREM), which includes 3 stages (N1, N2, and N3), rapid eye movement (REM) sleep, and amount of wake after initial sleep onset (WASO) [1]. Many studies have observed less NREM stage 3 (N3 or slow-wave sleep) and less REM in Black adults compared to white adults [2–10], which may be relevant to cardiometabolic health disparities. For example, experimental studies that suppressed N3, without changing the total amount of sleep, observed significantly increased blood pressure and reduced insulin sensitivity and glucose tolerance [11, 12], and these changes are risk factors for the development of hypertension and diabetes. Also, REM sleep reduction due to induced sleep fragmentation has been linked to reduced insulin sensitivity, as well as increased consumption of carbohydrates and fat, which pose a greater risk for diabetes and obesity [13–15]. Less REM sleep has been further associated with increased risk for heart failure and mortality [16, 17]. These diseases are disproportionately experienced by health disparity populations, such as Black adults, as well as individuals from lower socioeconomic groups. For decades, the Black population has been unfairly burdened by cardiometabolic diseases compared to non-Hispanic whites [18–22]. The importance of sleep health disparities was addressed by a 2020 workshop report [23] that emphasized the need for more research on the causes and consequences of sleep health disparities in order to better understand sleep’s role in other health disparities, and, importantly, identify strategies to mitigate the effects of these sleep disruptors.

Several studies have highlighted that neighborhood social and physical environments are interrelated with socioeconomic status and can affect health outcomes including sleep [23–27]. For example, people living in neighborhoods that have high crime rates and low social cohesion, poor housing conditions and dilapidated community structures are more susceptible to sleep disruptors and exhibit greater sleep problems [27–31]. One source of sleep disruption in the physical environment includes noise [32, 33], which can influence sleep and may play a role in sleep disparities [23]. Greater noise exposure in the home has been associated with greater self-reported insomnia symptoms and sleep disturbances, self-reported poor sleep quality, and actigraphy measured lower sleep efficiency, higher wake after sleep onset (WASO), and greater fragmentation index [32, 34–37]. The American Academy of Sleep Medicine survey in 2023, showed that outside noise was one of the most common sleep disruptors among US adults [38]. However, these prior studies did not examine the contribution of noise to sleep health disparities.

The sound pressure level of normal conversation is approximately 60 decibels (dB) and up to 70 dB has been considered to be safe during waking hours [39]. Sustained exposure to loud noise of varying intensity can lead to immediate hearing loss or long-term health consequences, including cardiovascular disease, cancer, and neurodegenerative diseases [32]. These negative health effects may be partially explained by disturbed sleep [32, 40, 41]. Animal research found that noise exposure during the sleep phase led to oxidative stress induced vascular and brain damage, circadian misalignment, and endothelial dysfunction, while daytime noise exposure was reported to have small or no effect [32, 42]. In humans, inflammation, increased adrenaline release, and endothelial dysfunction were reported after a single night of aircraft noise exposure [43]. A meta-analysis of 36 observational studies showed that average nighttime noise levels > 40 decibel (dB), especially from transportation sources (airplane, bus, and train), were associated with greater subjective sleep disturbance [33]. Additionally, several laboratory-based experimental studies have shown adverse effects of noise on sleep architecture [44–46], however, very little is known about noise level and its effect on sleep architecture in a real-world setting outside the laboratory. If we are to develop methods to improve the sleep of populations experiencing health disparities, we need to identify the sources of these sleep disparities, and we hypothesize that sound may be one such source. Therefore, our goal was to examine whether sound levels could explain differences in sleep architecture between Black and White adults.

The aims of these analyses included, first, to test whether sleep architecture and objectively measured sound levels varied by two important sociodemographic factors: race (non-Hispanic black vs non-Hispanic white) and education, as one indicator of socioeconomic status. Second, we examined whether sound levels in the bedroom were associated with measures of sleep architecture assessed on the same night. Our hypotheses were that sound levels would be higher in the bedrooms of the Black adults and those with lower education and that higher sound levels would be associated with worse sleep (i.e., less N3, less REM, more wake).

## Methods

### Study Design

These data were from two studies conducted between 2011 and 2016 that used identical methodology to assess sleep architecture and sound levels in the home. Participants in both studies were volunteers from the community. Study 1 recruited any adult participant (> 18 years) who had previously had a polysomnography recording (PSG) at the University of Chicago research laboratory or sleep clinic. The only exclusion criterion was acute illness. Study 2 recruited adult volunteers between the ages of 21–50 years who identified as non-Hispanic black or non-Hispanic white, and exclusion criteria included diabetes, sleep disorders, history of cardiovascular event(s), medication use, drug abuse, color blindness, Lasik eye surgery, women who were post-menopausal, and shift work. For these analyses we applied the following inclusion criteria: self-identify as non-Hispanic black or white (there were insufficient numbers of participants in other groups from Study 1), ages between 21–50 years (to be consistent with Study 2), and who had an apnea–hypopnea index (AHI) less than 15 events per hour (to avoid confounding due to moderate-severe sleep disordered breathing). The final sample size was 54 (15 from Study 1 and 39 from Study 2). Institutional Review Board approval was obtained prior to the initiation of both studies and all subjects provided written informed consent.

### Measures

#### Sleep Architecture

Sleep architecture was assessed with unattended in-home polysomnography (PSG) in both studies. Home PSG has good agreement with in-lab PSG sleep characteristics [47–49] and is more ecologically valid since sleep is recorded in their own homes. On one night, research personnel visited the participant’s home in the early evening to set up the ambulatory PSG machine (Track-It, Nihon Kodhen, Irvine, CA). The PSG recordings in both studies included electroencephalography (EEG), electrooculography (EOG), electromyography (EMG), and electrocardiography (ECG), all of which were used to identify specific sleep stages. In study 1, airflow was also measured using a nasal pressure transducer and thermocouples, respiratory effort was recorded from thoracic and abdominal respiratory inductance plethysmography, and pulse oximetry was measured continuously. Participants in study 2 had a laboratory PSG prior to the home PSG that included these respiratory measures, which was used to exclude those with AHI ≥ 15 events per hour. Hypopnea was calculated as ≥ 3% reduction in oxygen saturation or an arousal [50]. The sleep data was scored in 30-s epochs as stage *Wake, N1, N2, N3 or REM* by a trained and experienced rater blinded to the subjects’ demographics using standard criteria [51, 52]. *Percentages of N2, N3, and REM* were computed as minutes of each stage divided by total sleep time.

#### Sound

During the night of the in-home PSG, a Larson Davis SoundTrack LxT was placed next to the participant’s bed. The sound meter measured average sound pressure levels in decibels using A-weighting and a slow detector setting over 60-s intervals. Raw data was downloaded and exported into Microsoft Excel using the Larson Davis SLM-G3 software, which organized the sound measurements into 15-min bins. By cross-referencing the sleep start and end times determined by the PSG data, in-bedroom sound levels were examined only during each subject’s unique nocturnal sleep period. We calculated (1) the *average sound level throughout the entire sleep period* and (2) the *number of times maximum sound levels in each 15-min bin were above 60 dB.* For reference, 60 dB is approximately the sound level of normal conversation [39]. In addition, we calculated (3) t*he average sound level in the first 3 h of the sleep period* for comparison with N3, since N3 predominantly occurs in the first half of the sleep period.

#### Sociodemographic Factors

A sociodemographic questionnaire was administered and included questions about self-identified race, gender, and education level. The education question specifically asked, “What is the highest grade (or year) of regular school you have completed?” and responses options were presented as Elementary School: 01, 02, 03, 04, 05, 06, 07, 08; High School: 09, 10, 11, 12; College: 13, 14, 15, 16; Graduate School: 17, 18, 19, 20 +. Participants selected the year/grade corresponding to the highest one completed. The median in this sample was approximately 16 years so we dichotomized at “College degree or less” or “More than a college degree”.

### Statistical Analysis

Continuous variables are described using mean and standard deviation (SD) and categorical variables with number and proportions. Our first set of analyses involved univariate analysis to compare the sleep and sound measures between the two racial groups and two education groups using student t tests. Our second set of analyses involved linear regression models to examine the association between sound levels and sleep variables. We estimated both unadjusted models and models adjusted for age and gender. We also generated scatterplots of the associations between sound levels and PSG measures, including Pearson’s Correlations values for illustrative purposes. Finally, we estimated the extent to which sound levels were associated with the racial or socioeconomic sleep disparity by calculating the percent reduction in the race or education coefficient estimate following adjustment for sound levels [53]. Specifically, we first tested for associations between sleep stages and race or education group after adjusting for age and gender (Model 1) and then we added the sound measure to Model 1 for those models in which the sleep measure differed significantly between the racial or education groups. All tests were two-tailed, and significance was considered at p < 0.05. Statistical analyses were performed using StataSE (v14).

## Results

The analytical sample included 54 adults (based on the inclusion criteria stated above) between the ages of 21 and 49 years, 57% were women and 48% were Black adults (Table 1). The median educational level was 16 years so we dichotomized at college degree or less (59% of sample) and more than a college degree (41%).

**Table 1:** Description of the full sample and by race and education

	Full Sample (N = 54)	Race groups		Education Groups	
		Non-Hispanic black adults (n = 26)	Non-Hispanic white adults (n = 28)	College degree or less (n = 32)	More than a college degree (n = 22)
	**Mean (SD) or N (%)**				
***Demographic Variables***					
Age	31.1 (8.1)	30.8 (8.4)	31.4 (7.9)	30.3 (8.1)	32.3 (8.2)
Women, n (%)	31 (57.4%)	26 (48.2%)	28 (51.9%)	32 (59.3%)	22 (40.7%)
Total AHI	4.2 (4.2)	4.3 (4.2)	4.1 (4.2)	4.0 (4.2)	4.4 (4.2)
***Sleep Variables***					
Sleep Onset (hours)	23.5 (1.6)	23.7 (1.9)	23.4 (1.3)	23.4 (1.4)	23.8 (1.8)
Time in Bed (hours)	7.5 (1.2)	7.2 (1.2)	7.8 (1.3)	7.6 (1.2)	7.4 (1.4)
Total Sleep Time (hours)	7.0 (1.1)	6.7 (0.9)	7.3 (1.2)*	7.0 (1.0)	6.9 (1.3)
N2 duration (min)	235.7 (60.6)	240.1 (59.2)	231.6 (62.6)	247.0 (61.1)	219.1 (57.2)
N3 duration (min)	59.5 (32.0)	47.9 (29.5)	70.3 (30.9)*	52.6 (36.8)	69.5 (20.2)
REM duration (min)	97.2 (39.4)	80.3 (29.2)	112.9 (41.6)*	94.3 (43.2)	101.5 (33.7)
WASO (min)	30.2 (33.0)	34.9 (36.6)	25.7 (29.3)	35.5 (39.3)	22.4 (19.3)
N2 (%)	56.1 (11.5)	59.5 (10.2)	52.9 (11.9)*	58.5 (12.6)	52.5 (8.8)
N3 (%)	14.5 (7.9)	12.5 (8.1)	16.3 (7.3)	12.6 (8.8)	17.2 (5.3)*
REM (%)	22.8 (7.3)	20.1 (6.6)	25.4 (7.0)*	21.9 (8.3)	24.2 (5.3)
***Sound Variables***					
Average sound level – entire night (dB)	42.7 (9.9)	44.5 (10.4)	41.0 (9.3)	44.8 (10.3)	39.6 (8.7)
Average sound level—first 3 h (dB)	41.0 (10.7)	44.2 (11.1)	38.1 (9.6)*	43.5 (11.2)	37.4 (9.0)*
No. of sound peaks > 60 dB	6.0 (7.4)	8.0 (9.6)	4.1 (3.7)*	7.4 (9.0)	3.9 (3.3)

*p <.05 based on t test comparing either race groups or education groups

### Sleep Architecture and Sound Levels by Social Factors (Race, Education)

Table 1 presents the means for the sleep architecture measures by racial groups and by education groups. On average, Black participants had significantly shorter total sleep time, less N3, less REM, higher N2% and lower REM% than the White participants in this sample, however average sleep onset time and time in bed did not differ significantly. The only significant difference between the education groups in this sample was for N3% where those with a college degree or less had a lower N3%.

**Fig. 1:**
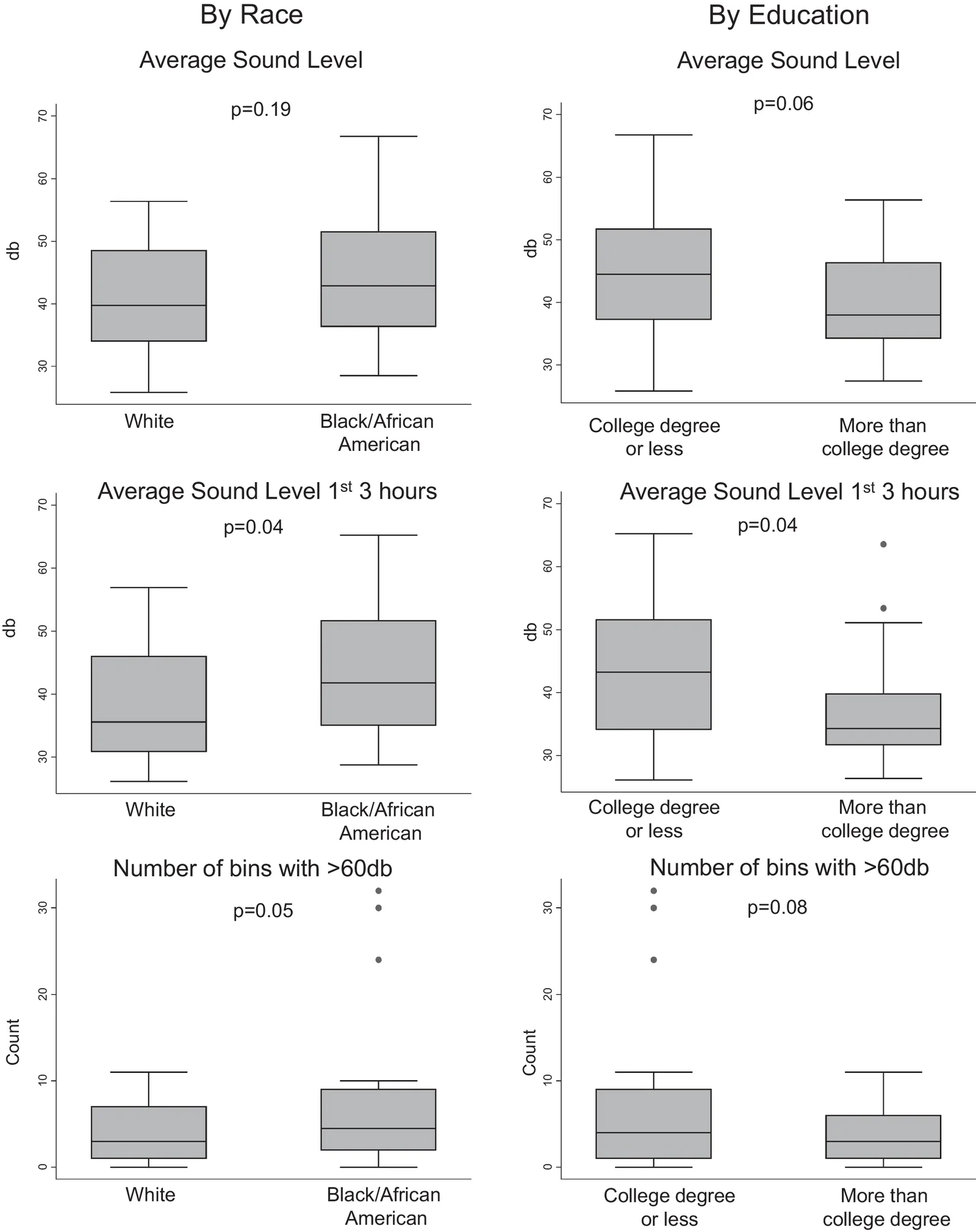
Box and whisker plots presenting the distribution of average sound levels by race and by education groups. *P* values are from Student’s *t* tests comparing the two groups

Sounds levels differed significantly between the racial groups (Table 1, Fig. 1); sounds levels were higher on average in the bedrooms of the Black participants compared to White participants, particularly in the first 3 h of the sleep period. Mean number of sound peaks > 60 dB was also significantly higher in the bedrooms of the Black participants compared to White participants. The average sound level during the first 3 h of the sleep period were significantly lower in the higher education group.

### Associations Between Sound Levels and Sleep Architecture

**Fig. 2:**
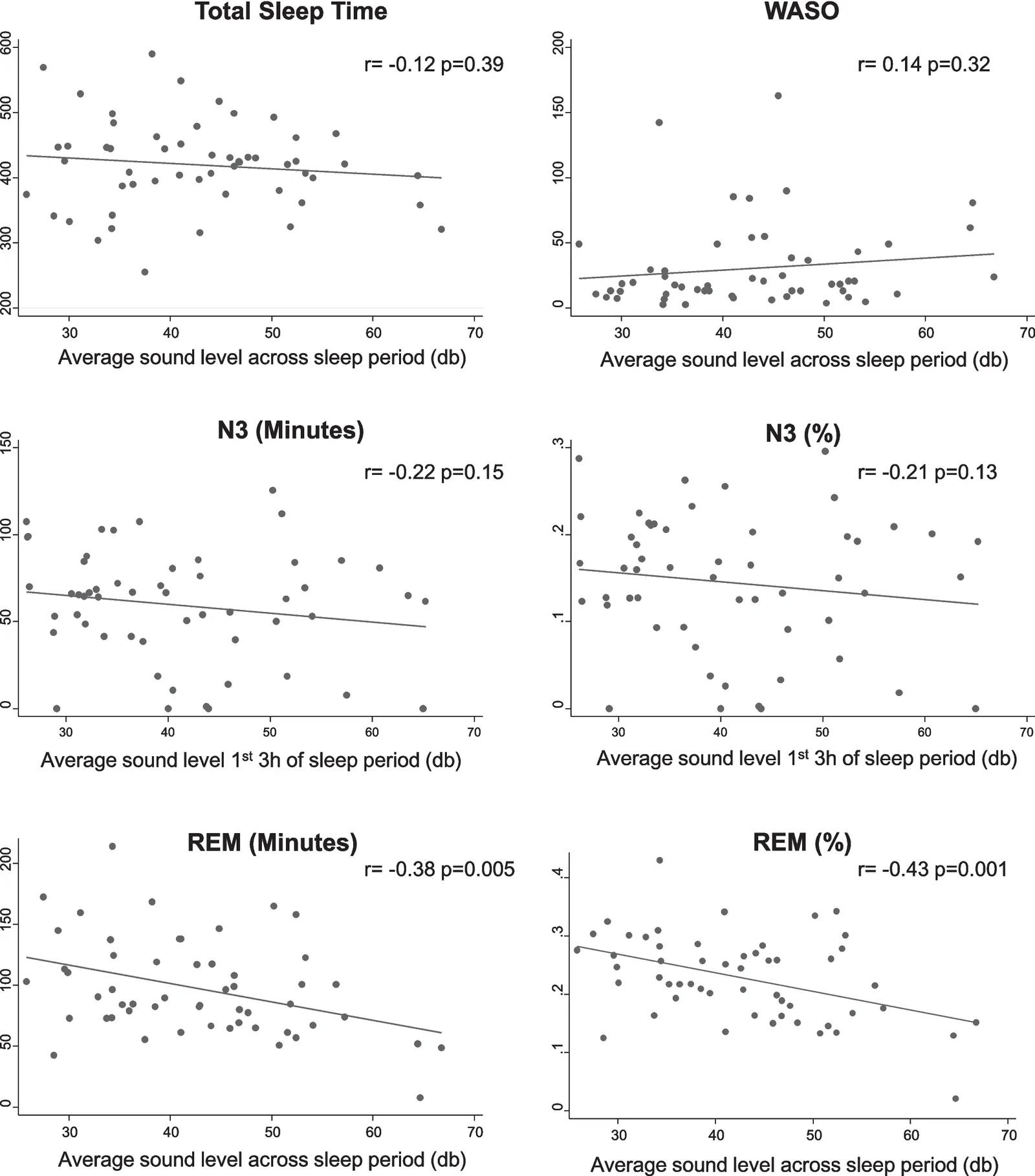
Unadjusted associations between average sound level during the sleep period and sleep characteristics. Pearson’s *r* value and associated *p* values are presented for each association

The unadjusted associations between average sound level and sleep characteristics from PSG are presented in Fig. 2 and Table 2. These analyses indicate that greater average sound levels during the entire sleep period and the number of sound peaks > 60 dB were all significantly associated with less REM sleep and lower REM%. Average sound levels during the entire sleep period were also significantly associated with higher N2%. Sound levels were not significantly associated with TST, N2 minutes, N3 minutes or % or WASO.

**Table 2:** Association between sound levels and sleep architecture variables

Outcome	Exposure—Average sound level entire night			
	Model 1		Model 2	
	β (95% CI)	*p*-value	β (95% CI)	*p*-value
Total sleep time (hours)	−0.82 (−2.70,1.07)	0.39	−0.72 (−2.70, 1.25)	0.47
N2 (minutes)	0.18 (−1.40, 1.75)	0.82	0.24 (−1.38, 1.86))	0.77
N3 (minutes) ^a^	−0.51 (−1.33, 0.30)	0.21	−0.54 (−1.37, 0.28)	0.19
REM minutes	−1.51 (−2.53, −0.49)	**0.005**	−1.50 (−2.58, −0.42)	**0.008**
WASO (minutes)	0.46 (−0.45, 1.39)	0.32	0.51 (−0.41, 1.44)	0.27
N2 (%)	0.40 (0.10,0.71)	**0.009**	0.43 (0.12, 0.75)	**0.007**
N3 (%) ^a^	−0.10 (−0.30, 0.10)	0.32	−0.11 (−0.31, 0.10)	0.28
REM (%)	−0.32 (−0.50, −0.13)	**0.001**	−0.32 (−0.51, −0.12)	**0.002**
**Outcome**	**Exposure- Number of sound peaks > 60 dB**			
	Model 1		Model 2	
	β (95% CI)	*p*-value	β (95% CI)	*p*-value
Total sleep time (hours)	−1.0 (−3.57, 1.50)	0.42	−0.90 (−3.50, 1.70)	0.49
N2 (minutes)	0.74 (−1.53, 3.01)	0.52	0.74 (−1.59, 3.07)	0.53
N3 (minutes) ^a^	−0.79 (−2.0, 0.40)	0.19	−0.70 (−1.90, 0.49)	0.24
REM minutes	−1.67 (−3.07, −0.25)	**0.02**	−1.61 (−3.06, −0.16)	**0.03**
WASO (minutes)	1.09 (−0.11, 2.30)	0.08	1.02 (−0.17, 2.23)	0.09
N2 (%)	0.31 (−0.11, 0.73)	0.14	0.30 (−0.13, 0.73)	0.17
N3 (%) ^a^	−0.16 (−0.45, 0.13)	0.29	−0.14 (−0.44, 0.150)	0.32
REM (%)	−0.36 (−0.61, −0.10)	**0.006**	−0.36 (−0.622, −0.09)	**0.008**

Model 1: unadjusted

Model 2: adjusted for age and gender

^a^N3 estimates (both minutes and %) used average sound level for first 3 h

When adjusting for age and gender, greater average sound levels across the entire night remained significantly associated with fewer minutes of REM sleep, higher N2% and lower REM%. A larger number of sound peaks > 60 dB also remained significantly associated with fewer minutes of REM sleep and lower REM%. None of the other sleep stages were significantly associated with sound levels in the adjusted models (Table 2).

Finally, we estimated the proportion of the racial and educational differences in sleep explained by sound levels (Table 3). In the age- and gender-adjusted models, Black participants had significantly less total sleep, less N3, less REM, and lower REM percentage than the White participants. Sound levels explained approximately 2.4% of the difference in total sleep time, 6.0% of the difference in N3 minutes, 14.5% of the difference in REM minutes and 21.1% of the difference in REM percentage. Participants with more than a college degree had significantly more N3 sleep and higher N3 percentage and sound levels explained approximately 10.6% and 6.0%, respectively, of these differences.

**Table 3:** Differences in sleep measures by race and by education and the percentage explained by sound levels

	Racial Groups		Education Groups	
	**Model 1**	**Model 2**	**Model 1**	**Model 2**
**Total Sleep Time (minutes)**	−41.8 (−78.3, −5.3)	−40.8 (−78.7, −2.9)	−5.3 (−44.1, 33.6)	n/a
% attenuation	**–**	2.4%	**–**	**–**
**N2 (minutes)**	7.9 (−26.5, 42.3))	n/a	−29.0 (−62.9, 5.0)	n/a
% attenuation	**–**	**–**	**–**	**–**
**N3 (minutes)**	−23.4 (−40.0, −6.9)	−22.0 (−39.6, −4.4)	18.9 (1.7, 36.2)	16.9 (−1.2, 35.1)
% attenuation	**–**	6.0%	**–**	10.6%
**REM (minutes)**	−35.1 (−55.1, −15.1)	−30.0 (−49.9, −10.2)	8.4 (−14.1, 30.9)	n/a
% attenuation	**–**	14.5%	**–**	**–**
**N3 (%)**	−4.0 (−8.3, 0.3)	n/a	5.0 (0.7, 9.2)	4.7 (0.1, 9.2)
% attenuation	**–**	**–**	**–**	6.0%
**REM (%)**	−5.7 (−9.5, −1.9)	−4.5 (−8.2, −0.9)	2.4 (−1.7, 6.5)	n/a
% attenuation	**–**	21.1%	**–**	**–**

All values are reported as estimate (95% CI) for either the race coefficient (Black v. White) or education coefficient (more than a college degree vs less education) in models for each sleep outcome

Only models where the sleep measure significantly differed by race or by education are included in Model 2

Model 1 includes racial or education group, age, and sex. Model 2 adds average sound level across the night (except for N3/N3%, which used average sound level for first 3 h) to Model 1

Percent attenuation is calculated as the percent reduction in the race coefficient estimate relative to the estimate in Model 1

## Discussion

We observed higher sound levels in the bedrooms of the Black participants than in the bedrooms of the White participants, and the Black participants had significantly worse sleep architecture than the White participants. There were fewer differences between the two education groups. Those with higher education (i.e., more than a college degree) had lower average sound levels during the first 3 h of the sleep period, and a higher percentage of N3. Further, higher sound levels were significantly associated with less REM sleep (minutes or percentage) and higher N2 percentage. Finally, sound levels explained a larger percentage of the racial differences in REM sleep and REM percentage (14.5 and 21.1%, respectively) than differences in total sleep or N3, which is consistent with the finding that sound levels were most strongly associated with REM sleep.

Our finding that Black participants had worse sleep based on less NREM Stage 3 and REM is consistent with other studies of sleep disparities. For example, studies from the SWAN sleep cohort of women in midlife (mean age 50 years) observed less NREM Stage 3 sleep in the Black women compared to the White women [8, 54]. Other observational studies also observed less NREM Stage 3 in Black adults compared to White adults [2, 3, 7]. A meta-analyses combined 14 US studies to compare the sleep of Black and White adults [10], and found that Black adults had significantly shorter TST, lower N3 percentage but higher REM percentage. Other studies have observed significantly worse sleep health among individuals of lower socioeconomic status as well [55, 56]. One study examined sleep using PSG in relation to socioeconomic status (based on education and income) and found that higher SES was associated with less WASO but was not associated with REM, N3 or TST [7]. We did not observe many differences in the PSG measures between our education groups, however, our sample had a high level of education, on average. Future work should examine the role of noise in impaired sleep quality among lower socioeconomic groups. Together, these studies, including ours, indicate worse sleep quality based on sleep architecture in Black adults and adults of lower socioeconomic status, which is why we examined noise/sound as one potential explanation for this racial disparity.

Other studies have demonstrated a detrimental impact of noise or sound levels on sleep. One experimental study observed several detrimental changes on PSG characteristics after 3 weeks of noise administered during sleep for four nights per week. These effects included greater WASO and decreased TST, N3 and REM-sleep [57]. We only observed an association with REM sleep, which may be due to the single night of measurement and the real-world assessment where noise levels varied more between participants. Also, the source of sound could be different among these participants, and a previous study suggested that transition from sleep stages to wake due to noise depend on its source [58]. Another experimental study tested four different transportation noises, each of a single night, on PSG measures in 26 young adults (19–33 years) and 16 older (52–70 years) adults but observed no effects on the PSG sleep stages [44]. They did, however, observe greater frequency of arousals in the older adults [44]. Our sample only included adults ≤ 50 years so our results may not be generalizable to older adults whose sleep may be more easily disrupted. One observational study examined sound levels based on a georeferenced map of sound levels and found a significant association between higher sound levels and self-reported short sleep duration [59], however this study did not measure sound inside the bedroom nor did they have PSG measures. A case–control study compared the sleep of individuals living near an airport (n = 46, 22–77 years) to those who did not (n = 32, ages 22–68 years) [41]. Although mean actigraphy-measured sleep fragmentation was higher in the group living near the airport, it was not statistically significant. To our knowledge, ours is the first study to use an objective measure of sound levels in a real-world setting, inside the participant’s bedroom, and while PSG was recorded.

Our finding is important given that the REM sleep is associated with several health domains. As described above, less REM sleep has been associated with cardiometabolic disease risk, including reduced insulin sensitivity, increased consumption of carbohydrates and fat, and heart failure and mortality [13–17]. Additionally, REM sleep is accompanied by activation of the autonomic nervous system; acceleration in heart rate during transition to REM sleep is linked to increased prevalence of fatal ventricular arrhythmias and sudden cardiac death [60]. Noise induced changes in sleep stages may compound the risk of fatal events as a consequence of autonomic responses [61]. While studies have shown > 40dB of sound can be detrimental to sleep and health [33, 40], it is also important to note that the average sound level during sleep in our study was > 40 dB, particularly in Black participants and among those with less education, whose mean sound level during sleep was approximately 45 dB. Further, REM sleep also plays an important role in learning and emotional memory processing, and less REM sleep can increase risk of impaired cognitive performance and impaired mood regulation [62, 63]. Moreover, a prior experimental study showed that sound administered during REM sleep resulted into altered cognitive performance [64].Taken together, the reduced amount of REM sleep observed in Black participants has important implications for cardiometabolic and cognitive health.

Our analyses suggested that sound levels may play a role in racial differences in REM sleep. We did not find other published studies of sound as a mediator of sleep disparities; however, a few studies have tested other potential mediators of racial sleep disparities. One study examined racial discrimination as a potential mediator for racial differences in insomnia severity in 1,458 individuals (74% white,19% Black) [65]. They reported that racial discrimination did significantly mediate the relationship between race (White vs Black) and insomnia symptom severity, explained approximately 61% of the association between race and insomnia symptom severity. Discrimination and psychological distress were also examined as potential mediator for self-reported sleep health in a sample of 7,749 adults (17% Black, 83% White) from the Health Retirement Study (HRS). In serial mediation analyses, discrimination and depressive symptoms explained nearly 6.8%, discrimination and loneliness explained about 4.3%, and discrimination and chronic stress explained about 3.6% of the association between race and self-reported poor sleep health [66]. Psychological distress also mediated the associations between neighborhood characteristics, crime rate and social cohesion, and sleep health in a large sample (n = 2699) from two low-income, predominately Black neighborhoods [31]. Finally, another study tested whether neighborhood disadvantage mediated racial differences in actigraphy-assessed sleep and found that neighborhood disadvantage explained approximately 24% of the race difference in WASO [29]. No single mediator will explain completely racial or socioeconomic disparities in sleep; there are numerous potential socioecological factors that impact sleep, from local environmental factors to structural racism [23].

There are strengths to the present study, including the objective measure of sound conducted in the bedroom of the participant on the same night that PSG was recorded. However, there are some important limitations, including the cross-sectional design, which does not allow for causal interpretations. We employed convenience sampling, which could raise possibility for selection bias, thus limiting the generalizability of study findings. Also, this sample is, on average, highly educated (the median was a college degree), and therefore findings related to education may not be generalizable to other populations. Future work should examine the impact of noise on sleep in groups of lower socioeconomic status. Another limitation to this study is that the study sample sizes were not determined a priori to test these associations. However, we performed power analyses using our sample’s standard deviation and sample size. We estimated that a sample of 54 participants provides approximately 85 percent power to detect an association of at least 1.25 fewer minutes of N3 per decibel of sound in the first 3 h (or 12.5 fewer minutes per 10 dB). We would have reduced power to detect weaker associations. Finally, we cannot determine the source of the sound (e.g. internal, external) in the bedrooms, which would be important to identify as we move towards developing mitigation strategies.

## Conclusion

These data suggest that sound levels are higher on average in the bedrooms of the Black adults compared to White adults, which could partially explain differences in sleep quality, particularly REM sleep. Jackson et al. [23] previously identified environmental factors as determinants of social disparities in sleep (e.g., among races and ethnicities or among socioeconomic groups). Future intervention studies that mitigate the effect of sound should be tested as a strategy to improve sleep, and should include assessment of efficacy, feasibility and acceptability. Mitigating the effects of sound may be a novel strategy to improve sleep health in these groups.

## Data Availability

The data that support the findings of this study are available from the corresponding author upon reasonable request.
